# *CSMD1* as a causative gene of developmental and epileptic encephalopathy and generalized epilepsies^☆^

**DOI:** 10.1016/j.gendis.2024.101473

**Published:** 2024-11-29

**Authors:** Wenjun Zhang, Sheng Luo, Mi Jiang, Yongxin Chen, Rongna Ren, Yunhong Wu, Pengyu Wang, Peng Zhou, Jiong Qin, Weiping Liao

**Affiliations:** a Department of Neurology, Institute of Neuroscience, Key Laboratory of Neurogenetics and Channelopathies of Guangdong Province and the Ministry of Education of China, The Second Affiliated Hospital, Guangzhou Medical University, Guangzhou, Guangdong 510000, China; b School of Medical Laboratory, Shao Yang University, Shaoyang, Hunan 422000, China; c Department of Pediatrics, Guangdong General Hospital, Guangzhou, Guangdong 510000, China; d Department of Pediatrics, The 900 Hospital of the Joint Service Support Force of the People's Liberation Army of China, Fuzhou, Fujian 350000, China; e Department of Neurology, Children's Hospital of Shanxi, Taiyuan, Shanxi 030000, China; f Department of Pediatrics, Peking University People's Hospital, Beijing 100044, China

**Keywords:** *CSMD1*, Developmental and epileptic encephalopathy, Genotype-phenotype correlation, Idiopathic generalized epilepsy, Minor allele frequency-phenotype severity correlation

## Abstract

Genetic factors are the major causes of epilepsies, such as developmental and epileptic encephalopathy (DEE) and idiopathic generalized epilepsy (IGE). However, the etiology of most patients remains elusive. This study performed exon sequencing in a cohort of 173 patients with IGE. Additional cases were recruited from the matching platform in China. The excess and damaging effect of variants, the genotype-phenotype correlation, and the correlation between gene expression and phenotype were studied to validate the gene–disease association. *CSMD1* compound heterozygous variants were identified in four unrelated cases with IGE. Additional *CSMD1* variants were identified in five cases with DEE featured by generalized seizures from the matching platform, including two with *de novo* and three with compound heterozygous variants. Two patients were refractory to antiseizure medications and all patients were on long-term therapy. The *CSMD1* variants presented a significantly high excess of variants in the case-cohort. Besides *de novo* origination, the DEE cases had each of the paired variants located closer to each other than the IGE cases or more significant alterations in hydrophobicity. The DEE-associated variants were all absent in the normal population and presented significantly lower minor allele frequency than the IGE-associated variants, suggesting a minor allele frequency-phenotype severity correlation. Gene expression analysis showed that *CSMD1* was extensively expressed throughout the brain, particularly in the cortex. The *CSMD1* temporal expression pattern correlated with the disease onset and outcomes. This study suggests that *CSMD1* is associated with epilepsy and is a novel causative gene of DEE and generalized epilepsies.

## Introduction

Epilepsy is one of the most common neurological diseases in children, characterized by recurrent seizures, which are classified into focal and generalized seizures.^1^ Patients with focal seizures may have a variety of acquired etiologies, such as structural, infectious, and immune causes.^2^ In contrast, generalized epilepsies generally have no acquired causes and are believed to be genetically determined.^3^ Previously, 2946 genes have been reported to be associated with epilepsy/seizures.^4^ However, only 21 genes were potentially causative/susceptible genes of generalized epilepsies, including *CACNA1H*, *CACNB4*, *CASR*, *CLCN2*, *EFHC1*, *GABRA1*, *GABRB3*, *GABRD*, *HCN2*, *HCN4*, *ICK*, *KCNMA1*, *MARCH6*, *RAPGEF2*, *RORB*, *SAMD12*, *SLC12A5*, *SLC2A1*, *STARD7*, *TNRC6A*, and *YEATS2* (OMIM database; www.omim.org)^4^; and the gene–disease associations in serval genes, such as *CACNA1H*, *CACNB4*, and *EFHC1* were even considered being disputed.^5^, ^6^, ^7^ The etiologies in most cases with generalized epilepsies remain elusive.^8^ On the other side, 112 genes have been associated with developmental and epileptic encephalopathy (DEE), which only explains the etiologies of approximately 30% of patients.^9^^,^^10^ Additionally, overlapped causative genes were presented in generalized epilepsies and DEE, such as *GABRA1*,^11^^,^^12^ *GABRB3*,^13^^,^^14^ and *SLC2A1*.^8^^,^^15^^,^^16^

In this study, a trio-based whole-exome sequencing approach was conducted on a cohort of 173 individuals diagnosed with generalized epilepsy. *CSMD1* compound heterozygous variants were identified in four unrelated cases with idiopathic generalized epilepsy (IGE). Additional *CSMD1* variants were identified in five cases with DEE featured by generalized seizures from the matching platform, including two with *de novo* and three with compound heterozygous variants. The gene–disease association of *CSMD1* was supported by the high excess of variants, the genotype-phenotype correlation, and the evidence of gene expression profile. This finding indicates that *CSMD1* is a potentially novel causative gene contributing to DEE and generalized epilepsies.

## Materials and methods

### Subjects

A cohort of 173 cases with generalized epilepsy was recruited over five years, from 2018 to 2022, at the Epilepsy Centre of Guangzhou Medical University Affiliated Second Hospital. All the patients had no acquired cause. Clinical data was collected from the affected individuals, including birth history, seizure onset age, seizure type/frequency, responses to anti-seizure medication, family history, neurological and general examination results, and diagnosis.^17^^,^^18^ Long-term video-electroencephalograph monitoring records were performed to detect epileptic discharges. The electroencephalograms were analyzed by two qualified electroencephalographers. Magnetic resonance imaging of the brain was conducted to detect structure abnormality. Diagnosis and classification of epileptic seizures and syndromes adhered to the criteria of the Commission on Classification and Terminology of the International League Against Epilepsy (1989, 2001, 2010, 2017, and 2022). All enrolled patients underwent a minimum follow-up period of one year.

To verify the clinical repetitiveness of *CSMD1* variants in patients with epilepsy, additional *CSMD1* variants in epilepsy patients were collected from the Matching Platform of the China Epilepsy Gene 1.0 Project (https://epg1.cn/).^19^ Patients with acquired causes or pathogenic variants of known epilepsy-associated genes were excluded.

This study was approved by the Ethics Committee of The Second Affiliated Hospital of Guangzhou Medical University under reference number 2020-h5-49, and written informed consent was obtained from the legal guardians of the patients.

### Whole-exome sequencing

Blood samples were obtained from the probands, their respective parents, and other available family members, and genomic DNA was extracted facilitated through the QIARamp Blood Mini Kit (Qiagen, Hilden, Germany). Trio-based whole-exome sequencing was performed on a HiSeq 2000 system (Illumina, San Diego, CA, USA), following the detailed sequence procedures described in previous studies.^2^^,^^20^^,^^21^ The sequencing data were produced through high-throughput parallel sequencing, average depth over 100 times, and comprehensive cover area over 98% of the targeted capture regions, thus providing high-quality sequencing outcomes. The raw sequencing data were mapped to the Genome Reference Consortium Human Genome build 37 (GRCh37) using the Burrows-Wheeler alignment. Single-nucleotide variants and insert/deletion variants were identified and annotated using the Genome Analysis Tool Kit.

### Genetic analysis

A case-by-case approach was used to identify candidate pathogenic variants in each case. Primarily, the rare variants with a minor allele frequency (MAF) below 0.005 in the Genome Aggregation Database (GnomAD, gnomad.broadinstitute.org) were prioritized.^22^^,^^23^ Then, potentially pathogenic variants were retained, including canonical splice site, frameshift, initiation codon, in-frame, nonsense, and missense, as well as intronic and synonymous variants predicted to affect splicing. The potentially pathogenic variants within each trio were analyzed using an individualized approach.^24^ Variants were filtrated according to the inheritance origin of each trio. Within each trio, variants with explainable genetic origins were selected. Then, a tiered MAF criterion was utilized to filter the variants: the MAF of *de novo*, hemizygous, and homozygous variants was set to absent in the control population of gnomAD; for compound heterozygous variants, the product of the frequencies of the two alleles in gnomAD was required to be less than 1 × 10^−6^, which is seven times less than the probability of observing an individual with such genotype in the current gnomAD population (1/141456 = 7 × 10^−^^6^). Variants were finally filtrated, based on information on each gene summarized in Genetic Dependence & Pathogenicity Database (www.gdap.org.cn), by criteria on the gene profile of four aspects.

i) The epilepsy candidate pathogenic genes should be expressed in the brain (inclusion criteria), and the possibility of other explainable pathogenic mechanisms should be carefully considered, such as ectopic expression of abnormal metabolites and long-term toxicity.
ii) The pathogenic genes that have been previously defined in gene–disease associations (genotype-phenotype associations) were excluded and the possibility of epilepsy as a phenotype was eliminated (exclusion criteria).
iii) The probability of being intolerant to heterozygous/homozygous variants of loss-of-function (pLI/pRec), genes of pLI ≥0.9 with *de novo* variants, and genes of pLI ≥0.9/pRec ≥0.9/pNull ≤0.1 with recessive variants were considered.
iv) Whether gene knockout/knockdown conditions produce relevant brain phenotypes was investigated.

The *CSMD1* gene is one of the candidate genes that fit the screening criteria, with recurrently identified bi-allelic and *de novo* variants in this cohort. Sanger sequencing was applied to verify the candidate pathogenic variants. All the *CSMD1* mutations identified in this study were annotated in the reference transcript NM_033225000.

### Excess analysis of *CSMD1* variants

The excess of *CSMD1* in the case-cohort was assessed by recessive burden analysis^25^ and aggregate frequency of variants. For the burden of recessive variants, the observed number of recessive *CSMD1* variants was compared with the expected number by chance in the East Asian populations, to exclude the occurrence occasionality of recessive *CSMD1* variants in the case-cohort. For aggregate frequency analysis of identified variants, the aggregate frequency of the *CSMD1* variants in the case-cohort was compared with that of controls, including the control of general and the East Asian populations in the gnomAD database.^23^ To analyze the association between MAF and phenotypic severity, the MAF of IGE- and DEE-associated variants were compared with that of “benign/likely benign” variants (from the NCBI-Gene database, https://www.ncbi.nlm.nih.gov/gene).

### Protein modeling

Iterative Threading ASSEmbly Refinement software (I-TASSER, https://zhanglab.ccmb.med.umich.edu/I-TASSER/) was used for protein modeling. PyMOL (version 1.7; Schrödinger, LLC; NY, USA) was used to visualize and analyze molecular structural alterations. The I-Mutant Suite server (folding.biofold.org/cgi-bin/i-mutant2.0.cgi) was used to predict the changes in the stability of variant proteins, represented by alterations in free energy (ΔΔG, kcal/mol).

### Expression analysis of *CSMD1* gene

The RNA expression of *CSMD1* in tissues was analyzed by the data of the Human Protein Atlas dataset (HPA; www.proteinatlas.org) and Genotype-Tissue Expression (GTEx, www.gtexportal.org) dataset. The expression levels of the top 15 tissues were visualized. The Brainspan database (http://www.brainspan.org/) from 8 post-conceptional weeks to 40 years was used to explore the spatio-temporal expression pattern. The expressional spline is fitted by the locally weighted scatterplot smoothing (LOWESS) algorithm to interpret the expression pattern of *CSMD1*.

### Statistical analysis

Data analysis was performed with the R statistical software (version 4.0.2). A two-sided Fisher exact test, as recommended by ClinGen,^26^ was employed to compare the frequency of *CSMD1* gene variants between the epilepsy group and the control group. The Mann–Whitney test was used to analyze the difference in MAF among varied phenotypes. A *p* value < 0.05 was considered statistically significant.

## Results

### Identification of *CSMD1* variants in patients

**Figure 1:**
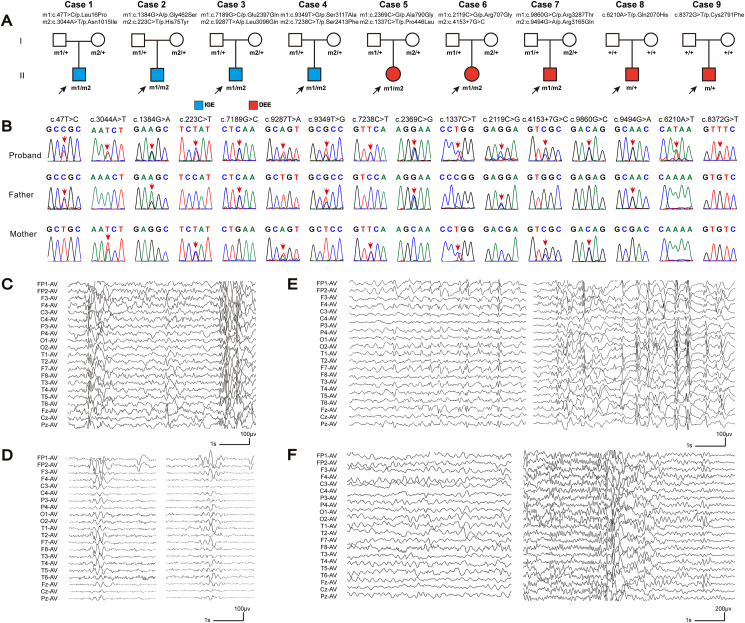
Genetic data and representative EEGs of cases with *CSMD1* variants. **(A)** Pedigrees of the cases with *CSMD1* variants and their corresponding phenotypes. **(B)** DNA sequence chromatogram of the *CSMD1* variants. The arrows indicate the positions of the variants. **(C)** Interictal EEG of case 1 at 6 years old showed generalized polyspikes, polyspike-slow waves, and multifocal spike waves. **(D)** Interictal EEG of case 2 at 20 years old showed generalized spike-slow waves, with dominance in the frontal lobe. **(E)** Interictal EEG of case 3 at 7 years old showed multifocal spike-slow waves and irregular generalized spike-slow waves. **(F)** EEG of case 6 at 15 years old showed slow waves in the background (left) and interictal generalized spike-slow waves (right). EEG, electroencephalogram.

Four pairs of *CSMD1* compound heterozygous variants were identified in four unrelated cases (cases 1, 2, 3, and 4) with IGE from the Epilepsy Centre of the Second Affiliated Hospital of Guangzhou Medical University, including c.47T > C/p.Leu16Pro & c.3044A > T/p.Asn1015Ile, c.223C > T/p.His75Tyr & c.1384G > A/p.Gly462Ser, c.7189G > C/p.Glu2397Gln & c.9287T > A/p.Leu3096Gln, and c.7238C > T/p.Ser2413Phe & c.9349T > G/p.Ser3117Ala (Fig. 1A and Table 1).

**Table 1:** Clinical features of the patients with *CSMD1* variants.

Case	Variants (NM_033225.5)	Gender	Age	Seizure onset	Seizure course	Effective ASMs	Seizure freeduration	EEG	Brain MRI	Development	Diagnosis
Case 1	c.47T > C/p.Leu16Pro c.3044A > T/p.Asn1015Ile	Male	13 yr	3 yr	GTCS 4 times/day; clonic and myoclonic twice/day; FS (1.5–2yr).	VPA, TPM, LTG	8 yr	Generalized spike-slow and poly-spike waves (6 yr).	Normal	Normal	IGE
Case 2	c.223C > T/p.His75Tyr c.1384G > A/p.Gly462Ser	Male	21 yr	18 yr	GTCS twice; FS (1yr).	VPA	2 yr	Generalized spike and spike-slow waves; predominantly in the frontal lobe (20 yr).	Normal	Normal	IGE (GTCA)
Case 3	c.7189G > C/p.Glu2397Gln c.9287T > A/p.Leu3096Gln	Male	5 yr	3 yr	GTCS twice; CPS 2–3 times/week; FS (2yr).	VPA, LTG, LEV	Refractory	Irregular generalized spike, spike-slow, and poly-spike-slow waves; occasionally multifocal spike wave (5 yr).	Normal	Normal	IGE
Case 4	c.7238C > T/p.Ser2413Phe c.9349T > G/p.Ser3117Ala	Male	19 yr	8 yr	GTCS 1–4 times/mo; myoclonic once/month in 14 yr.	VPA	6 yr	Generalized spike and spike-slow (15 yr).	Normal	Normal	IGE (JME)
Case 5	c.1337C > T/p.Pro446Leu c.2369C > G/p.Ala790Gly	Female	6 yr	9 mo	Spasm 3–4 times/day.	VPA, TPM	2 yr	Generalized sharp and spike-slow waves; bilateral frontal, temporal, and central areas sharp and spike-slow waves (6 yr).	Normal	Mild ID	DEE
Case 6	c.2119C > G/p.Arg707Gly c.4153+7G > C	Female	9 yr	6 yr	Tonic 5–6 times/day; atypical absence once.	VPA, LTG, LEV	Refractory	Generalized 1.5–2.5 Hz spike, spike-slow, and poly-spike-slow waves (7 yr).	Normal	Mild ID	DEE
Case 7	c.9494G > A/p.Arg3165Gln c.9860G > C/p.Arg3287Thr	Male	6 yr	1 yr	Myoclonic 3–4 times/day; atypical absence 2 times/mo.	VPA, CLB	3 yr	Generalized 2–2.5 Hz sharp-slow waves (2 yr); bilateral central, and temporal areas sharp and sharp-slow waves, predominantly in left.	Delayed myelination in posterior ventricular horns?	Mild ID	DEE
Case 8	c.6210A > T/p.Gln2070His	Female	4.5 yr	6 mo	Spasm 6–7 times/day.	LEV	NA	Generalized sharp and sharp-slow waves, predominantly in the frontal lobe (8 mo).	Normal	GDD	DEE
Case 9	c.8372G > T/p.Cys2791Phe	Male	9 yr	6 yr	GTCS twice; myoclonic or atonic 7–8 times/day.	VPA, LTG	NA	Generalized spike-slow and poly-spike-slow waves (7 yr).	Normal	Mild ID	DEE

Abbreviations: ASMs, anti-seizure medication; CLB, Clobazam; CNZ, clonazepam; DEE, developmental and epileptic encephalopathy; EEG, electroencephalogram; FS, febrile seizure; GDD, global developmental delay; GTCA, epilepsy with generalized tonic-clonic seizure; GTCS, generalized tonic-clonic seizure; JME, juvenile myoclonic epilepsy; LEV, levetiracetam; LTG, lamotrigine; mo, month (s); MRI, magnetic resonance imaging; NA, not available; ID, intellectual disability; IGE, idiopathic generalized epilepsy; TPM, topiramate; VPA, valproate; yr, year (s).

Two missense variants p.Asn1015Ile and p.Ser3117Ala were absent in the population of the GnomAD database, and the remaining six variants presented extremely low frequencies (MAF <2.09 × 10^−4^; Table 2). The aggregation frequencies of *CSMD1* variants identified in this cohort were found to be significantly higher than those in the gnomAD population, including both the gnomAD-all population (*p* = 1.96 × 10^−13^) and the control of the gnomAD-all population (*p* = 3.08 × 10^−12^). When the recessive variant burden analysis was analyzed,^25^ the number of recessive *CSMD1* variants identified in this cohort was significantly higher than would be expected by chance in the East Asian population (MAF <0.005; *p* = 1.45 × 10^−3^).

**Table 2:** Aggregate frequency of *CSMD1* variants identified in this study.

Identified *CSMD1* Variants		Allele Count/Numberin this study	Allele Count/Numberin the gnomAD-allpopulation	Allele Count/Numberin the controls ofgnomAD-all population	Number ofHomozygotesin the controlsof gnomAD
Variants from the epilepsy centre of the Second Affiliated Hospital of Guangzhou Medical University					
Case 1	c.47T > C/p.Leu16Pro	1/346	3/277942 (0.00001079)	2/117540 (0.00001702)	0
	c.3044A > T/p.Asn1015Ile	1/346	−/−	−/−	–
Case 2	c.223C > T/p.His75Tyr	1/346	11/249270 (0.00004413)	7/108530 (0.00006450)	0
	c.1384G > A/p.Gly462Ser	1/346	2/245928 (0.000008132)	1/106842 (0.000009360)	0
Case 3	c.7189G > C/p.Glu2397Gln	1/346	6/246494 (0.00002434)	3/107214 (0.00002798)	0
	c.9287T > A/p.Leu3096Gln	1/346	43/278862 (0.0001542)	25/119392 (0.0002094)	0
Case 4	c.7238C > T/p.Ser2413Phe	1/346	1/245634 (0.000004071)	1/106754 (0.000009367)	0
	c.9349T > G/p.Ser3117Ala	1/346	−/−	−/−	–
Total		8/346	66/245634(0.00026869)	39/106754 (0.0036533)	0
*p* Value			1.964 × 10^−13^	3.083 × 10^−12^	
OR (95% CI)			88.2459 (36.21832–186.61225)	64.73943(25.91799–142.13550)	

Variants from China epilepsy Gene V.1.0 matching platform					
Case 5	c.1337C > T/p.Pro446Leu		−/−	−/−	–
	c. 2369C > G/p.Ala790Gly		−/−	−/−	–
Case 6	c.2119C > G/p.Arg707Gly		1/31388 (0.00003186)	−/−	0
	c.4153+7G > C		−/−	−/−	–
Case 7	c.9494G > A/p.Arg3165Gln		−/−	−/−	–
	c.9860G > C/p.Arg3287Thr		−/−	−/−	–
Case 8	c.6210A > T/p.Gln2070His		−/−	−/−	–
Case 9	c.8372G > T/p.Cys2791Phe		−/−	−/−	–

*p* values and ORs were estimated with two-sided Fisher's exact test.

Abbreviations: gnomAD, Genome Aggregation Database.

***CSMD1***: RefSeq transcript NM_033225.5.

Additional *CSMD1* variants, including two *de novo* variants (c.6210A > T/p.Gln2070His and c.8372G > T/p.Cys2791Phe) and three pairs of compound heterozygous variants (c.2369C > G/p.Pro446Leu & c.1337C > T/p.Ala790Gly, c.2119C > G/p.Arg707Gly & c.4153+7G > C, and c.9494G > A/p.Arg3165Gln & c.9860G > C/p.Arg3287Thr), were identified in five unrelated cases (cases 5, 6, 7, 8, and 9) from the matching platform in China (Fig. 1A and Table 1). The five cases presented with DEE featured generalized seizures. The variants were all absent in the controls of the gnomAD database.

No pathogenic or likely pathogenic variants of known epilepsy-associated genes were identified in the nine patients.^27^

### Damage effects of *CSMD1* variants

**Figure 2:**
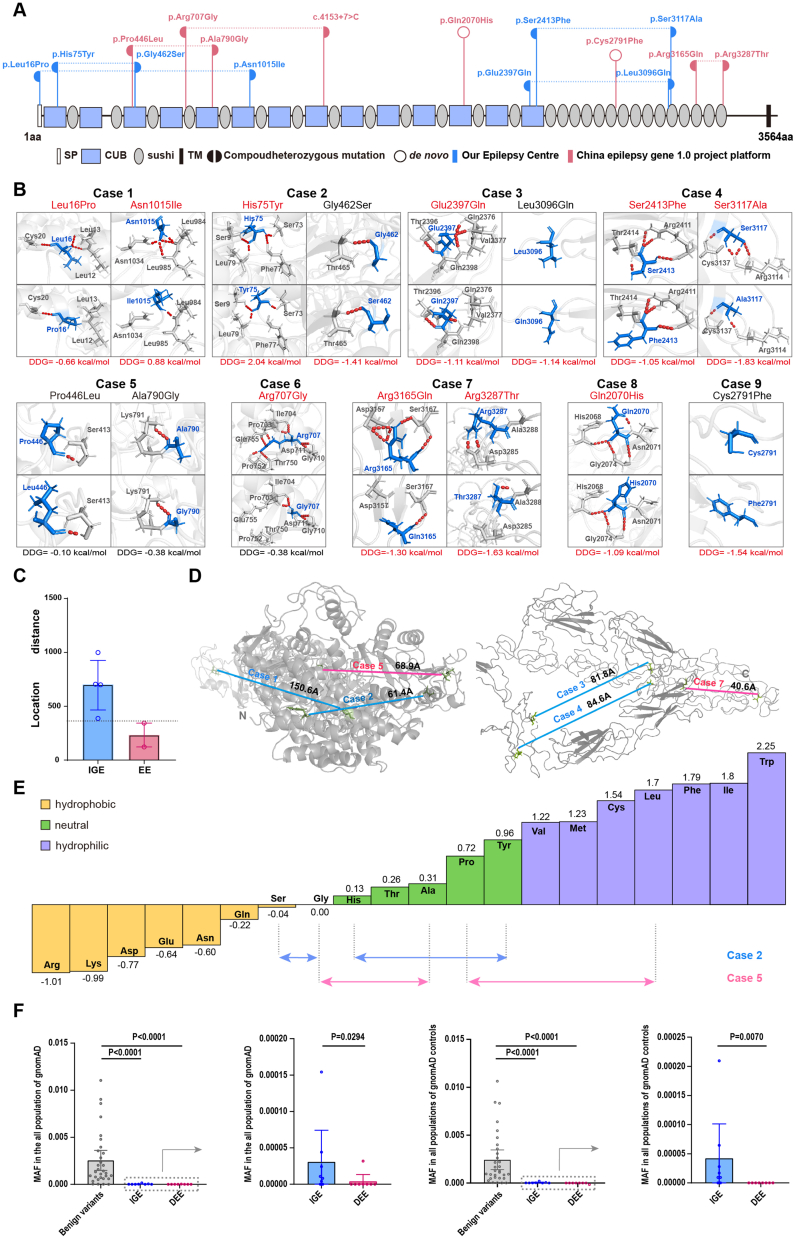
Schematic illustration of the variant locations, hydrogen bond changes, and MAF-phenotype correlations of *CSMD1* variants. **(A)** Schematic diagram of the CSMD1 protein and the localization of the *CSMD1* variants identified in this study. **(B)** Hydrogen bond changes and ΔΔG values of *CSMD1* variants. A positive or negative ΔΔG value indicates abnormally reduced or enhanced mutant protein stability, respectively. The red dotted line represents hydrogen bonds. **(C)** Hydrophobicity of amino acids calculated by the Fauchère and Pliska hydrophobicity scale. Amino acids with high positive values are more hydrophobic, whereas amino acids with low negative values are more hydrophilic. Patient 5 (DEE) exhibited obvious hydrophobicity changes compared with patient 2 (IGE) (0.31 plus 0.98 versus 0.04 plus 0.83). **(D)** Comparison of the two-dimensional distances of two variants in each biallelic missense variant. **(E)** Schematic of spatial (three-dimensional) distances in *CSMD1*. The red line indicates DEE patients, while the blue line indicates IGE patients. **(F)** The MAF comparison between benign variants and variants associated with IGE/DEE. The aggregate frequency of IGE- and DEE-associated variants were compared, with the “benign/likely benign” variants from the NCBI-Gene database (https://www.ncbi.nlm.nih.gov/gene) serving as controls. MAF, minor allele frequency; DEE, developmental and epileptic encephalopathy; IGE, idiopathic generalized epilepsy.

The CSMD1 protein consists of a signal peptide, 14 CUB domains, 28 sushi domains, and a short cytoplasmic tail.^28^ The identified variants were all located in functional domains (including one in the signal peptide) (Fig. 2A).

The molecular effect of missense variants was analyzed with protein modeling using I-TASSER and visualization using PyMOL. It was predicted that ten missense variants would alter hydrogen bonds with the surrounding residues (Fig. 2B). The variants p.Gly462Ser, p.Cys2791Phe, and p.Leu3096Gln did not alter the hydrogen bonds between the protein and surrounding residues but were predicted to significantly decrease protein stability. All the missense variants were predicted to be damaged by at least one *in silico* tool (Table 3).

**Table 3:** Genetic features of the individuals with *CSMD1* variants.

Case	Coordinate	Nucleotide change	Protein change	Inheritance	MAF	SIFT	PP2_Var	Mutation Taster	CADD	FATHMM_MKL	GERP	phyloP	phastCons	SiPhy
Case 1	Chr8: 4851892	c.47T > C	p.Leu16Pro	Paternal	1.702 × 10^−5^	D (0.05)	B (0)	P (1)	D (21.6)	T (0.171)	NC (−1.39)	NC (0.364)	NC (0.919)	NC (7.574)
	Chr8: 3224625	c.3044A > T	p.Asn1015Ile	Maternal	–	D (0.003)	PD (1.0)	DC (1)	D (28.1)	D (0.972)	C (5.09)	C (6.023)	C (1.000)	C (14.856)
Case 2	Chr8: 4494943	c.223C > T	p.His75Tyr	Maternal	6.45 × 10^−5^	T (1.0)	B (0.107)	P (0.998)	T (10.42)	D (0.934)	C (5.12)	C (5.293)	C (1.000)	C (16.054)
	Chr8: 3351209	c.1384G > A	p.Gly462Ser	Paternal	9.36 × 10^−6^	T (0.016)	PD (0.996)	DC (1)	D (28.8)	D (0.975)	C (5.48)	C (7.722)	C (1.000)	C (18.131)
Case 3	Chr8: 2949134	c.7189G > C	p.Glu2397Gln	Paternal	2.798 × 10^−5^	T (0.341)	B (0.013)	P (1)	T (7.823)	T (0.337)	C (4.87)	NC (1.372)	NC (0.067)	C (14.88)
	Chr8: 2820911	c.9287T > A	p.Leu3096Gln	Maternal	2.094 × 10-^4^	T (0.307)	PD (0.462)	P (1)	T (11.26)	T (0.466)	C (4.87)	NC (0.636)	NC (0.453)	NC (4.536)
Case 4	Chr8: 2949085	c.7238C > T	p.Ser2413Phe	Maternal	9.367 × 10^−6^	D (0)	PD (0.999)	DC (1)	D (32)	D (0.992)	C (5.8)	C (9.707)	C (1.000)	C (20.042)
	Chr8: 2820849	c.9349T > G	p.Ser3117Ala	Paternal	–	T (0.28)	B (0.011)	P (0.999)	T (13.64)	T (0.278)	NC (−5.75)	NC (1.483)	NC (0.686)	C (21.130)
Case 5	Chr8:3432474	c.1337C > T	p.Pro446Leu	Maternal	–	D (0.022)	PD (0.992)	DC (1)	D (24.1)	D (0.990)	C (5.06)	C (9.764)	C (1.000)	C (17.210)
	Chr8: 3256949	c.2369C > G	p.Ala790Gly	Paternal	–	T (0.117)	PD (0.99)	DC (1)	D (23.6)	D (0.993)	C (5.35)	C (9.764)	C (1.000)	C (19.438)
Case 6	Chr8:3263696	c.2119C > G	p.Arg707Gly	Paternal	–	T (0.055)	PD (0.991)	P (0.590)	D (22.6)	D (0.791)	C (3.45)	C (2.478)	NC (0.929)	NC (11.858)
	Chr8:3141659	c.4153+7G > C	–	Maternal	–	–	–	–	–		–	–	–	–
Case 7	chr8: 2820122	c.9494G > A	p.Arg3165Gln	Maternal	–	T (0.543)	B (0.313)	P (1)	T (10.83)	T (0.123)	C (2.85)	NC (0.353)	NC (0.000)	NC (5.474)
	Chr8: 2813245	c.9860G > C	p.Arg3287Thr	Paternal	–	T (0.507)	PD (0.985)	DC (0.999)	T (16.93)	D (0.964)	C (5.64)	C (3.894)	NC (0.999)	NC (10.764)
Case 8	Chr8: 3000018	c.6210A > T	p.Gln2070His	*de novo*	–	D (0.005)	PD (0.89)	DC (0.785)	T (13.61)	D (0.841)	NC (−4.41)	NC (0.109)	NC (0.965)	C (12.342)
Case 9	Chr8: 2855538	c.8372G > T	p.Cys2791Phe	*de novo*	–	D (0.015)	PD (1.0)	DC (1)	D (34)	D (0.989)	C (6.07)	C (7.729)	C (1.000)	C (20.644)

Abbreviations are as follows: B, benign; C, conserved; CADD, Combined Annotation Dependent Depletion; D, damaging; DC, disease-causing; GERP, Genomic Evolutionary Rate Profiling; MAF, the minor allele frequency of general population from the Genome Aggregation Database (controls); NC, non-conserved; PD, probably_damaging; PP2, Polyphen2_HVAR; SIFT, Sorting Intolerant From Tolerant; P, polymorphism; T, tolerable.

### Clinical features of the patients with *CSMD1* variants

The clinical features of the patients with *CSMD1* variants are summarized in Table 1.

The patients in cases 1, 2, 3, and 4 were diagnosed as IGE, including epilepsy with generalized tonic-clonic seizure alone in case 2 and juvenile myoclonic epilepsy in case 4. Their ages of seizure onset were preadult, ranging from 3 years to 18 years old. They presented with infrequent generalized seizures, including generalized tonic-clonic seizures, clonic seizures, and myoclonic seizures. Febrile seizures were observed in cases 1, 2, and 3. Seizure-free was achieved by valproic acid monotherapy (cases 2 and 4) or combined treatment including valproic acid medication (case 1). Their electroencephalograms showed generalized discharges, including generalized spike, spike-slow, and poly-spike waves (Fig. 1C–E). The four patients presented normal neurodevelopment. No brain structural abnormalities were detected by magnetic resonance imaging scans.

The five patients from the Matching Platform were diagnosed with DEE which was featured by generalized seizures and discharges on electroencephalograph recordings. Their onset ages of seizures ranged from 6 months to 6 years old. The seizures included spasms, atypical absence, generalized tonic-clonic seizures, and tonic, myoclonic, and atonic seizures. Seizures were frequent (daily) in the five cases (Table 1). The interictal electroencephalogram discharges included generalized sharp, spike/sharp-slow, poly-spike/sharp-slow, and multifocal sharp/sharp-slow waves (Fig. 1F from case 6 as a representative). The five patients all showed mild/borderline intellectual disability or global developmental delay. The magnetic resonance imaging scans detected no obvious abnormal brain structure, except possibly delayed myelination in the posterior area in case 7.

Notably, two patients with *CSMD1* variants were refractory to antiseizure medications and all patients were on antiseizure medications so far as we followed up.

### Genotype-phenotype correlation of *CSMD1* variants

Previous studies suggested that *de novo* variants were more common in patients with DEE.^29^ The two patients in this study with *de novo* variants presented with DEE. The other DEE cases presented compound heterozygous variants that did not differ from the variants of IGE in sub-molecular implications, including the location and alteration in hydrogen bonds/ΔΔGs (Fig. 2A, B). Previous studies indicated that the distance between the two variants of biallelic variants is associated with the severity of phenotypes.^2^^,^^30^^,^^31^ In this study, the variants of the paired biallelic variants in DEE cases (cases 5 and 7) were generally located closely in the secondary structure of the protein, compared with that of IGE cases (cases 1, 2, 3, and 4) (Fig. 2C). However, case 2 (IGE) had similar distance with case 5 (DEE) in secondary structure, as well as the spatial structure (Fig. 2D). Further analysis on hydrophobicity change showed that the two variants in case 5 were more significantly changed than the variants in case 2 (Fig. 2E), potentially explaining the severer phenotype of case 5.

### Correlation between MAF of *CSMD1* variants and phenotypic severity

The frequency of variants in populations is generally correlated with the phenotype prevalence,^32^ which is determined by the phenotype severity, potentially due to natural selection pressure. We thus studied the relationship between the MAF of the *CSMD1* variants and the phenotypic severity. The variants identified in the patients presented significantly lower MAF than the benign variants (*p* < 0.0001). The DEE-associated variants were all absent in the normal population and presented significantly lower MAF than the IGE-associated variants (*p* = 0.007), suggesting a correlation between MAF and phenotypic severity.

### Spatio-temporal expression of *CSMD1* and its association with phenotypes

**Figure 3:**
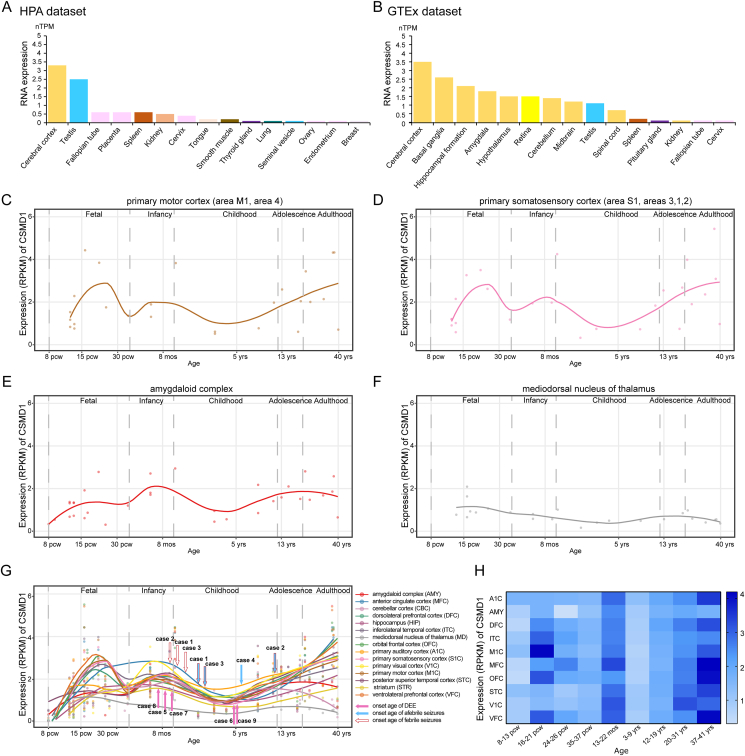
The spatio-temporal expression of *CSMD1* gene. **(A, B)** The top 15 tissues with high expression levels of the *CSMD1* gene. The *CSMD1* gene is extensively expressed in multiple brain regions, particularly the cerebral cortex, calculated by data obtained from the HPA and GTEx datasets. **(C**–**G)** The temporal expression pattern of *CSMD1* in diverse brain regions. The expression levels were retrieved from the human RNA sequencing data obtained from the BrainSpan database. The curve was fitted by the locally weighted scatterplot smoothing (LOWESS) method. **(H)** The heat map of the expression period of *CSMD1*. RPKM, reads per kilobase per million mapped reads; nTPM, normalized transcripts per million.

In this study, the patients all presented with generalized seizures. The spatial expression profile of *CSMD1* in the brain was analyzed to explore the possible molecular basis of generalized epilepsies. According to the HPA dataset, the *CSMD1* gene is predominantly expressed in the brain (Fig. 3A, B). The GTEx dataset showed in detail that *CSMD1* was extensively expressed in multiple brain regions, particularly the cerebral cortex, basal ganglia, hippocampal formation, amygdala, and hypothalamus, with the highest expression in the cerebral cortex.

Recent studies have shown that the genetic-dependent (expression) stage is associated with the evolutional process and onset age of diseases.^33^, ^34^, ^35^, ^36^ Thus, *CSMD1* temporal expression pattern was analyzed. The *CSMD1* was expressed highly in infancy, decreased in early childhood (with a nadir around four years old), but increased dramatically since later childhood (Fig. 3C–G), potentially being associated with the poor outcome/long-term antiseizure medication. The onset ages of patients were generally consistent with the temporal expression of *CSMD1* (Fig. 3G). Three of the DEE cases had onset at infancy, while the other two DEE cases had later onset (later childhood). One of the two later-onset cases (case 9) had the *de novo* heterozygous missense variant (c.8372G > T/p.Cys2791Phe) that potentially had a less damaging effect shown by lack of hydrogen bond change (Fig. 2B), compared with the *de novo* variant in case 8. Another childhood-onset DEE case (case 6) had one pair of biallelic variants containing one variant located close to the splice site (c.4153+7G > C), being potentially less damaging. These findings suggest that early onset was potentially associated with severely damaged variants. Three of the four cases with IGE had febrile seizures at early stages, suggesting that the precipitating factors (like fevers) potentially affect the onset age of the illness.

## Discussion

The *CSMD1* gene (OMIM∗ 608397) encodes CUB (complement C1r/C1s, Uegf, Bmp1) and sushi multiple domains protein 1,^28^ which acts as an inhibitor of C3 activation and cell clearance.^37^ C3 is involved in microglia-mediated synaptic pruning, and inhibition of C3 reduces the extent of early synapse loss.^38^^,^^39^ Additionally, it acts like neuropilin and neuronal growth cone proteins, playing a vital role in guiding axons toward synaptic targets and signal transduction during development.^40^ Homozygous *Csmd1* knockout mice exhibit neuropsychological deficits, including abnormal voluntary movement, anxiety, and despair behaviors,^41^ suggesting the important role of the *CSMD1* gene in neurodevelopment. However, the association between *CSMD1* and human diseases is undetermined. This study suggested that the *CSMD1* gene is a novel causative gene of DEE and generalized epilepsies.

Previously, *CSMD1* variants were occasionally detected in patients with epilepsy, including one *de novo* deletion in a family with four individuals affected by IGE with/without DD^42^ and one pair of compound heterozygous missense variants in a girl with epilepsy and bilateral polymicrogyria^43^ (Table 4). This study identified *CSMD1* variants in nine unrelated cases with epilepsy, including five with DEE and four with IGE. The *CSMD1* genes presented a significant excess of variants in the case-cohort. The gene–disease association was further supported by the phenotype–genotype correlation, the MAF-phenotypic severity correlation, and the spatial–temporal pattern of gene expression. A previous study indicated that the variants associated with severe phenotypes (such as DEE) tend to be absent or to present extremely low MAF as a control.^44^ This study provided direct evidence for the correlation between MAF and phenotypic severity, suggesting a potential role of MAF in evaluating the pathogenicity of variants.

**Table 4:** Previously reported *CSMD1* variants and phenotypes.

Case	Variants (NM_033225.5)	Original	MAF	Phenotypes	References
Case 1	c.5399T > A/p.Val1800Glu	*de novo*	–	ASD	Iossifov, O'Roak, et al. (2014)^56^
Case 2	c.3688C > T/p.Arg1230Cys	*de novo*	–	ASD	Iossifov, O'Roak, et al. (2014)^56^
Case 3	c.6761A > G/p.Gln2254Arg	*de novo*	–	ASD	Iossifov, O'Roak, et al. (2014)^56^
Case 4	c.1915G > A/p.Ala639Thr	*de novo*	–	ASD	Wang, Guo, et al. (2016)^57^
Case 5	c.3688C > T/p.Arg1230Cys	*de novo*	–	ASD	Wang, Guo, et al. (2016)^57^
Case 6	c.5399T > A/p.Val1800Glu	*de novo*	–	ASD	Wang, Guo, et al. (2016)^57^
Case 7	c.6761A > G/p.Gln2254Arg	*de novo*	–	ASD	Wang, Guo, et al. (2016)^57^
Case 8	c.2381A > C/p.His794Pro	*de novo*	–	ASD, ADHD	Guo, Duyzend, et al. (2019)^59^
Case 9	chr8:3812813_3957156del	*de novo*	–	ASD, DD	Saleh, Beyyumi, et al. (2021)^60^
Case 10	c.919G > A/p.Ala307Thr	*de novo*	–	DD	Turner, Wilfert, et al. (2019)^58^
Case 11	c.563G > A/p.Ser188Asn c.5344C > A/p.Gln1782Lys	Maternal	–	Epilepsy, cerebellar agenesis, polymicrogyria	Costanzo, Zanni, et al. (2022)^43^
		Paternal	–		
Case 12	chr8:4310831_4329349del	*de novo*	–	Epilepsy with/without DD	Naseer, chaudhary, et al. (2016)^42^
		Maternal			
		Maternal			
		Maternal			

Note: MAF, minor allele frequency; ADHD, attention deficit and hyperactivity disorder; ASD, autism spectrum disorder; DD, developmental delay.

DEE is a severe form of epilepsy with neurodevelopmental abnormalities. Currently, 112 genes have been associated with DEE (www.omim.org), explaining approximately 30% of patients.^9^^,^^10^ This study suggests *CSMD1* is potentially a novel causative gene of DEE. The patients with DEE in this study were featured by generalized seizures and generalized discharges on electroencephalograms, which was potentially a novel subtype of DEE. Such sub-classification implies a potential significance in identifying the causative genes of diseases^24^ and clinical management of patients.^36^

IGE is a common type of epilepsy, estimated to comprise 15%–20% of all epilepsy diagnoses.^45^^,^^46^ The etiology of IGE is generally attributed to genetic factors.^47^ However, only 21 genes have been identified as causative/susceptible genes of generalized epilepsies, among which the gene–disease associations in serval genes were even considered to be disputed.^5^, ^6^, ^7^ These genes could only explain a small portion of patients. Clinically, the majority of IGE patients (64%–85%) responded to antiseizure medication, but lifelong therapy is required in some patients.^48^, ^49^, ^50^ The mechanisms underlying the heterogeneous outcomes were not determined. This study suggested *CSMD1* as a novel causative gene of generalized epilepsies. Two patients with *CSMD1* variants exhibited refractory seizures and all patients were on long-term antiseizure medication. The poor outcomes could be viewed from the perspective of genetic-dependent (expression) stage.

Genes present distinct temporal expression patterns, which reflect how human life depends on the genes in different developmental stages, *i.e.*, the genetic-dependent stage.^34^ The age-dependent features of epilepsy are critical for the diagnosis and classification of epilepsy,^51^ which is the basis for further clinical management. Previously, the mechanism underlying the age-dependent features of epilepsy is unknown. Our recent studies suggested that the genetic-dependent (expression) stage is associated with phenotypic features, including the onset age, evolutional course, and outcomes.^33^^,^^35^^,^^36^^,^^52^^,^^53^ The present study suggested that the expression of *CSMD1* gene, which is high in infancy, decreased in early childhood, but increased dramatically since later childhood, is generally correlated with the onset age of the patients, indicating the genetic-dependent (expression) stage as one of the essential factors determining the disease onset age. Besides, damage of variants and precipitating factors were also determinants of onset age. Notably, the *CSMD1* gene is increasedly expressed since later childhood, potentially reflecting the increased functional demand/dependence for the gene, which may be one of the explanations for refractory seizures and an indicator of long-term therapy. The coincidence between the genetic-dependent (expression) stage and phenotypic features provided novel insights into disease development, being potentially helpful for genetic diagnosis and clinical management of epilepsy.

The *CSMD1* gene is highly expressed in the brain tissue, with the highest level in the cerebral cortex, which may provide the molecular basis for the phenotype of generalized seizures/discharges. A recent study showed that the knockdown of lectin pathway component C3 resulted in cortical lamination defects with ectopically placed cortical neurons.^54^ Dysfunction of *CSMD1* has been suggested to impact the C3 activation,^37^ potentially involved in corticogenesis. *CSMD1* knockout led to defects in the corticogenesis process, including neuroepithelium polarity and neural progenitor cell differentiation, in forebrain organoids differentiated from human embryonic stem cells.^55^ These findings suggested the role of *CSMD1* in neurodevelopmental disorders. However, further studies are required to determine the mechanisms underlying the generalized features of epilepsy and DEE.

This study has some limitations. Further experimental studies are required to determine the detailed functional alteration and pathogenic mechanism of *CSMD1* variants. Previous large-scale sequencing studies have detected several *de novo CSMD1* variants in patients with ASD/DD (Table 4).^56^, ^57^, ^58^ However, it was unknown whether those patients had seizures or not. Further studies are needed to determine the whole phenotypic spectrum of *CSMD1*. The outcomes of patients also need long-term follow-up.

In conclusion, this study suggested that *CSMD1* is associated with generalized epilepsies and is a novel causative gene of DEE. The gene–disease association is supported by the significant excess of variants, the phenotype–genotype correlation, the phenotypic severity-MAF correlation, and the spatial–temporal pattern of gene expression. Identification of *CSMD1* as a novel causative gene of DEE and the characteristics of *CSMD1*-associated DEE is potentially helpful for managing patients in clinical practice.

## Ethics declaration

The studies involving human participants were reviewed and approved by the Ethics Committee of The Second Affiliated Hospital of Guangzhou Medical University (No. 2020-hs-49), and written informed consent to participate in this study was provided by the participant's legal guardian/next of kin. The studies adhered to the guidelines of the International Committee of Medical Journal Editors with regard to patient consent for research or participation.

## CRediT authorship contribution statement

**Wenjun Zhang:** Writing – review & editing, Writing – original draft, Visualization, Validation, Software, Methodology, Investigation, Formal analysis, Data curation, Conceptualization. **Sheng Luo:** Writing – review & editing, Writing – original draft, Formal analysis, Conceptualization. **Mi Jiang:** Formal analysis, Data curation. **Yongxin Chen:** Data curation. **Rongna Ren:** Data curation. **Yunhong Wu:** Data curation. **Pengyu Wang:** Visualization, Formal analysis. **Peng Zhou:** Data curation. **Jiong Qin:** Data curation. **Weiping Liao:** Writing – review & editing, Writing – original draft, Project administration, Investigation, Funding acquisition, Conceptualization.

## Data availability

The data that support the findings of this study are available in the supplementary material of this article or available from the corresponding author upon reasonable request.

## Funding

This work was supported by the 10.13039/501100001809National Natural Science Foundation of China (No. 82271505 to W.P.L.), the Multi-center Clinical Research Fund Project of the Second Affiliated Hospital of Guangzhou Medical University (No. 010G271099, 2020-LCYJ-DZX-03 to W.P.L.), and the Science and Technology Project of Guangzhou, Guangdong, China (No. 201904020028 to W.P.L.). The funders had no role in study design, data collection and analysis, and the decision to publish or preparation of the manuscript.

## Conflict of interests

All authors claim that there is no conflict of interests.
